# Migraine prevalence in patients with atrial septal defect

**DOI:** 10.1186/1129-2377-14-63

**Published:** 2013-07-24

**Authors:** Yuji Kato, Takeshi Hayashi, Toshiki Kobayashi, Norio Tanahashi

**Affiliations:** 1 Department of Neurology and Cerebrovascular Medicine, Saitama International Medical Center, Saitama Medical University, 1397-1 Yamane, Hidaka, Saitama 350-1298, Japan; 2 Department of Pediatric Cardiology, Saitama International Medical Center, Saitama Medical University, 1397-1 Yamane, Hidaka, Saitama 350-1298, Japan

**Keywords:** Atrial septal defect, Right-to-left shunt, Migraine

## Abstract

**Background:**

Patients with a patent foramen ovale have a high prevalence of migraine with aura (MA). However, whether patients with an atrial septal defect (ASD) have a high prevalence of migraine remains unclear. The present study aimed to determine the prevalence of migraine and its clinical characteristics in patients with ASD.

**Findings:**

Ninety-five patients (age ≥ 20 years) who had undergone percutaneous ASD closure responded to a questionnaire used by neurologists to diagnose migraine either with or without aura. We diagnosed migraine before ASD closure according to the criteria of the International Headache Society. The overall prevalence of migraine seemed to be higher in the present study than in the Japanese general population (24.2% vs. 9.4%, respectively). All patients with MA were female and significantly younger than those without migraine (p < 0.01).

**Conclusions:**

Our findings suggested that the susceptibility to the development of MA differs according to sex and age in patients with cardiac shunt.

## Introduction

Patients with a patent foramen ovale (PFO) have a high prevalence of migraine with aura [1,2]. A right-to-left shunt (RLS) might be implicated in the pathogenesis of this disorder [3]. However, whether patients with atrial septal defect (ASD) have a high prevalence of migraine remains unclear. This might be because ASD is relatively rare (1–3 cases per 10,000 births) compared with PFO [4]. We recently reported the influence of percutaneous ASD closure on migraine and found *de novo* migraine in 11% of that study population [5]. In addition, a high prevalence of migraine was noted in that study before ASD closure [5]. In the present study, we focused on the effect of sex and age on the prevalence of migraine in patients with ASD and evaluated the clinical characteristics of migraine in these patients.

## Methods

### Patient selection

ASD was diagnosed by transesophageal echocardiography and cardiac catheterization. All patients aged ≥20 years who were included in our previous study and underwent transcatheter ASD closure at our center between August 2005 and March 2012 responded to a questionnaire that neurologists used to diagnose migraine either with or without aura (MA or MO, respectively). The questionnaire was constructed in accordance with the International Classification of Headache Disorders, 2nd edition [6]. The questionnaire was mailed to all patients from May 2012 to March 2013. The time interval from closure to questionnaire administration was 39 ± 19 months (range: 3 to 80 months). Migraine prevalence before ASD closure was compared with that reported in the Japanese general population [7], after adjustment for sex and age. The present study proceeded in accordance with our institutional ethics committee, and all patients and/or their relatives provided written, informed consent to participate in the study.

### Statistical analysis

Quantitative variables are expressed as means ± standard deviation, and qualitative variables are expressed as ratios (%). Age, sex, defect size and pulmonary-to-systemic blood flow ratio (Qp/Qs) were compared across groups using the Wilcoxon rank-sum test. Differences were considered statistically significant when p < 0.05. Data were analyzed using PASW Statistics software version 18 (SPSS, Inc., Chicago, IL).

## Findings

Among the 95 patients (response rate 79%) who participated in the study, the mean age was 45 ± 15 years and there were 67 females. The prevalence of migraine was 10.7% (0% MA and 10.7% MO) and 29.9% (9.0% MA and 20.9% MO) in men and women, respectively. The overall prevalence of migraine was 24.2% (6.3% MA and 17.9% MO). The 95% confidence interval (CI) of migraine prevalence ranged from 16.7% to 33.7%. Table 1 shows the prevalence of migraine in each age group for both sexes.

**Table 1:** Migraine prevalence by sex and age in patients with ASD (above) and published data for the general population in Japan (below)

**Sex**	**Age group**	**n**	**Migraine with aura**	**Migraine without aura**
**Male**	20-29	4	0 (0)	1 (25.0)
	30-39	3	0 (0)	2 (66.7)
	40-49	13	0 (0)	0 (0)
	50-59	4	0 (0)	0 (0)
	60-69	3	0 (0)	0 (0)
	70+	1	0 (0)	0 (0)
	**Total**	**28**	**0 (0)**	**3 (10.7)**
	95% CI	28	0-12.1	3.7-27.2
**Female**	20-29	11	2 (18.1)	2 (18.1)
	30-39	15	3 (20.0)	5 (33.3)
	40-49	9	1 (11.1)	0 (0)
	50-59	19	0 (0)	7 (36.8)
	60-69	10	0 (0)	0 (0)
	70+	3	0 (0)	0 (0)
	**Total**	**67**	**6 (9.0)**	**14 (20.9)**
	95% CI	67	4.2-18.2	12.9-32.1
**Total**		**95**	**6 (6.3)**	**17 (17.9)**
**95% CI**		95	2.9-13.1	11.5-26.8
**General population**				
**Male**		2,670	0.4*	2.4*
**Female**		3,070	1.3*	10.8*
**Total**		5,740	1.1*	8.3*

Values are shown as numbers (%). *Adjusted for sex and age distribution.

Abbreviation: *CI* confidence interval.

The study population was divided into groups consisting of patients without migraine and those with MA or MO, and their characteristics are shown in Table 2. Patients with MA were significantly younger than those without migraine (p < 0.01), and women tended to develop MA more frequently than men; however, the difference did not reach statistical significance.

**Table 2:** Characteristics of patients with and without migraine before ASD closure

**Characteristics**	**No migraine**			**Migraine**	**Migraine**
	**Total**	**Male**	**Female**	**With aura**	**Without aura**
**N**	72	25	47	6	17
**Age**	47.5 ± 14.7	46.4 ± 13.2	48.1 ± 15.6	32.5 ± 5.4*	41.1 ± 12.4
**Female sex, n (%)**	47 (65)	0 (0)	47 (100)	6 (100)	14 (82)
**ASD diameter (mm)**	19.5 ± 5.3	19.5 ± 6.3	19.4 ± 4.8	17.4 ± 7.4	19.4 ± 5.2
**Qp/Qs**	2.6 ± 1.3	2.4 ± 1.4	2.7 ± 1.2	2.9 ± 1.7	2.6 ± 0.9

*p < 0.01 vs. no migraine female; Qp/Qs, pulmonary to systemic blood flow ratio.

## Discussion

Patients with ASD had a 1.8- to 3.6-fold higher prevalence of migraine (24.2%; 95% CI, 16.7 - 33.7%) than the 9.4% found in the Japanese general population after adjusting for sex and age [7]. Compared with the Japanese general population, there was a 6.9-fold higher prevalence of MA in women, and a 4.5- and 1.9-fold higher prevalence of MO in men and women, respectively [7]. The group with MA was exclusively female and these patients were younger than females who did not have migraine. These findings suggest that susceptibility to MA differs according to sex and age in the presence of a cardiac shunt. The mechanism for a sex difference in migraine could be mediated by fluctuations in estrogen levels via their influence on cellular excitability or the cerebral vasculature [8]. The large CI in our sample represented uncertainty due to the small number of patients in each group.

The reported prevalence of migraine in patients with ASD ranges from 11 to 22% for MA and 12 to 28% for MO [9-12]. In contrast, we found a lower prevalence of MA (6.3%) but a similar prevalence of MO (17.9%) in our cohort of 95 patients. Although we found MA only in females, sex has never been reported to be associated with migraine type (MA or MO) in previous studies of patients with ASD. Mortelmans et al. reported a total migraine prevalence of 30% in ASD patients, and those with MA were older than patients without migraine [9]. They considered that MA in older patients could be associated with a right-to-left shunt (RLS) through an ASD, as they might be more prone to thrombus formation in the venous circulation [9]. In contrast, patients with MA were younger than those without migraine in the present study. This is similar to the age distribution of migraine patients in a population with PFO [13].

An ASD is mainly characterized by a left-to-right shunt. However, contrast echocardiography can detect an RLS during a Valsalva maneuver or exercise in patients with an ASD [14]. An RLS can also allow blood to bypass the lung filter. Microemboli and vasoactive chemicals such as serotonin might be potential migraine triggers. Alternatively, the inheritance of common genetic factors for cardiac defects and MA has been suggested. The common inheritance of atrial shunts including ASD with MA has been observed in some families [15].

In our previous study, we found an increase in the overall prevalence of migraine after ASD closure [5]. Nevertheless, in several patients, migraine disappeared after ASD closure [5]. Platelet aggregation possibly on the left side of the device or intra-atrial pressure imbalance after ASD closure might unmask a migrainous diathesis. Younger patients had a higher prevalence of migraine with aura before ASD closure, but some other patients developed *de novo* migraine after ASD closure.

Our study does have a few limitations. First, selection bias might be present because our patient population was selected from a cohort referred to our center for percutaneous ASD closure. Second, this retrospective study asked patients about their headache after ASD closure, and remembering the characteristics of headaches might have been difficult for some because of the long time interval (range: 3 to 80 months) from ASD closure to completion of the questionnaire. Therefore, recall bias might have been introduced. To minimize this potential confounding factor, we confirmed the occurrence of migraine in patients’ medical records. Third, we need to consider racial variations in migraine prevalence, as the prevalence of migraine is lower in Japan than in Western countries [16,17]. Fourth, our study lacked a control group and we compared our results with previously published results in the general Japanese population [7].

## Consent

Written informed consent was obtained from the patient for the publication of this report and any accompanying images.

## Abbreviations

PFO: Patent foramen ovale; ASD: Atrial septal defect; RLS: Right-to-left shunt; MA: Migraine with aura; MO: Migraine without aura.

## Competing interests

The authors declare that they have no competing interests.

## Authors’ contributions

YK conceived the project and drafted the manuscript. TK was responsible for ASD patients’ collection. TH and NT supervised the manuscript. All authors read and approved the final manuscript.
